# The complete mitogenome of the splendid japalure *Japalura splendida* (Squamata, Agamidae)

**DOI:** 10.1080/23802359.2019.1643797

**Published:** 2019-07-19

**Authors:** Weiyi Huang, Hongdi Luo, Sangdawa Luo, An Huang, Qingyong Ni, Yongfang Yao, Huailiang Xu, Bo Zeng, Ying Li, Zhimin Wei, Mingwang Zhang

**Affiliations:** a Farm Animal Genetic Resources Exploration and Innovation Key Laboratory of Sichuan Province, Sichuan Agricultural University, Chengdu, China;; b College of Animal Sciences and Technology, Sichuan Agricultural University, Chengdu, China;; c Forestry and Grassland Administration of Qushui County, Lhasa, China;; d College of Life Science, Sichuan Agricultural University, Ya’an, China;; e Institute of Millet Crops, Hebei Academy of Agriculture and Forestry Sciences, Shijiazhuang, China

**Keywords:** *Japalura splendida*, complete mitogenome, phylogeny

## Abstract

The complete mitogenome of *Japalura splendida* (16,673 bp in length) is determined and analyzed in this study. It contains 13 protein-coding genes, 2 rRNA genes, 22 tRNA genes, and one non-coding regions. All the genes in *J. splendida* are distributed on the H-strand, except for the *ND6* gene and seven tRNA genes which are encoded on the L-strand. The phylogenetic tree suggests that *J. splendida* and *Japalura flaviceps* formed a sister group and reveals the order ((((*Acanthosaura lepidogaster*, *Acanthosaura armata*), ((*J. splendida*, *J. flaviceps*), *Pseudocalotes microlepis*))), *Calotes versicolor*) with substantial support for the monophyly.

The splendid japalure, *Japalura splendida* (Barbour and Dunn, 1919) belongs to the family Agamidae, suborder Lacetilia and Oder Squamata. This lizard occurs only in China (Hunan, Hubei, Guizhou, Henan, Yunnan, Sichuan, Shanxi and Gansu provinces). In the last two decades, numerous mitogenomes of vertebrates have been sequenced and improved to obtain a reliable phylogeny (Zhang et al. 2003; Zhang et al. 2008; Wielstra and Arntzen 2011). Meanwhile, gene duplication, rearrangement, loss, and inversion of the vertebrate mitogenome have been reported (Rest et al. 2003; Amer and Kumazawa 2007; Zhuang and Cheng 2010; Liu et al. 2019). To further understand the evolution of mitogenome in lizards, we determined the complete mitogenome sequence of *J. splendida*.

The specimen of *J. splendida* was collected from pet market in Yunnan Province (N25°2′34″, E102°42′23″), China in 2018 and muscle tissue was taken and preserved in 100% ethanol. Total genomic DNA was extracted from the tissue and stored in −80 °C ultra-low temperature freezer using Ezup-pillar Genomic DNA Extraction Kit (Sangon, Shanghai, China). A DNA sample was shipped to Personal Biotechnology Co, Ltd (Shanghai, China) for library construction and sequencing by next-generation sequencing (NGS) methods (Metzker 2010). We analyzed the complete mitogenome of *J. splendida* and performed phylogenetic analysis for the new obtained and from the other lizard mitogenomes from the published sources.

The mitochondrial genome of *J. splendida* is 16,673 bp (GenBank No. MK940807) in length and contains 37 genes (13 protein-coding genes, 2 ribosomal RNAs, and 22 transfer RNAs genes) and 1 control region (D-loop). The overall nucleotide compositions of this mitogenome are A (34.88%), T (23.74%), C (28.02%), and G (13.35%), respectively with 58.63% A + T content. In this mitogenome, *ND6* gene and seven tRNA genes (*trnQ*, *trnA*, *trnN*, *trnC*, *trnY*, *trnS1*, and *trnE*) were encoded in the L-strand, while the rest of genes were encoded in the H-strand. The tRNAs varied from 53 to 74 bp in size, and the lengths of 12S rRNA and 16S rRNA were 843 and 1461 bp, respectively. In addition, the control region was 1482 bp long and is located between the tRNA-Pro and tRNA-Phe genes. The gene arrangement pattern and transcription directions were identical to previous studies on *Japalura flaviceps* (Liu et al. 2019).

Twenty-one mitogenome sequences including 20 Agamidae and one *Crocodylus palustris* were used for phylogenetic analyses. We selected the latter one as the outgroup species. Phylogenetic trees were performed by MEGA 6.0 using maximum-likelihood (ML) methods with the concatenated nucleotide sequences of 13 protein-coding genes (Tamura et al. 2013). The result of ML phylogenetic tree (Figure 1) shows that *J. splendida* and *J. flaviceps* formed a monophyletic group in the order ((((*Acanthosaura lepidogaster*, *Acanthosaura armata*)), ((*J. splendida*, *J. flaviceps*), *Pseudocalotes microlepis*))), *Calotes versicolor*) with high bootstrap support values (Figure 1). The phylogenetic relationships in Agamidae here conform to the results of a previous study (Liu et al. 2019).

**Figure 1.**
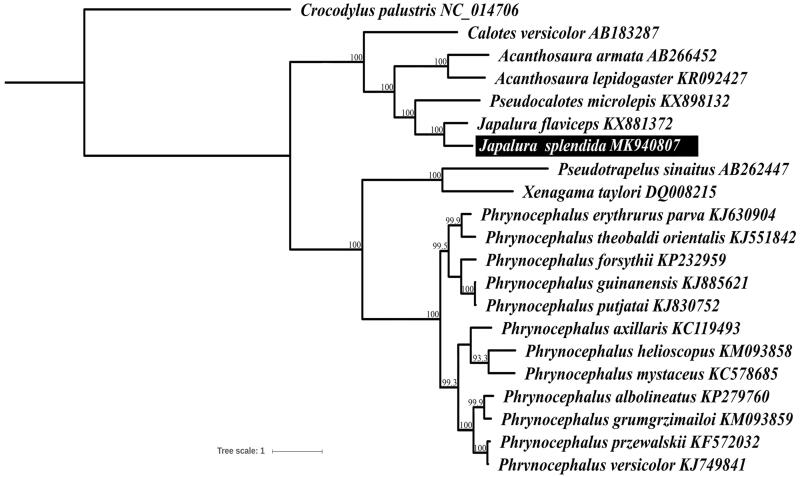
Maximum-likelihood (ML) phylogenetic tree of 21 reptile species based on 13 protein-coding genes. Bootstrap support values are shown at the nodes. The GenBank accession numbers are listed following species names.
